# Nutritional Value Improvement of Oats by Solid-State Fermentation with *Monascus purpureus*

**DOI:** 10.3390/foods14101703

**Published:** 2025-05-11

**Authors:** Yonghui Yu, Yingying Li, Jingjie Zhang, Jing Wang

**Affiliations:** 1 Key Laboratory of Geriatric Nutrition and Health, Ministry of Education, Beijing Technology and Business University, Beijing 100048, China; yonghuiwh@126.com (Y.Y.); liyingying_siren@163.com (Y.L.); 2 China-Canada Joint Lab of Food Nutrition and Health, Beijing Technology and Business University, Beijing 100048, China; 3 Key Laboratory of Special Food Supervision Technology, State Administration for Market Regulation, Beijing Technology and Business University, Beijing 100048, China

**Keywords:** oats, nutrients, phytochemicals, antinutritional factors, estimated glycemic index

## Abstract

With increasing research, the nutritional value of oats is gaining recognition. Fermentation is an emerging biotransformation pattern that changes the nutritional structure of whole grains. Currently, research on whole-grain fermentation is relatively focused on phenolic compounds and their antioxidants, but less attention has been given to avenanthramides (Avns) and the glycemic index (GI) in fermented oats. In this study, oats were subjected to solid-state fermentation (SSF) by *Monascus purpureus* for 21 days, and samples were taken at different time points. Changes in the contents of nutrients, phytochemicals, and antinutritional factors were analyzed using one-way ANOVA. Additionally, a simulated in vitro digestion experiment was conducted to assess the estimated glycemic index (eGI) of SSF oats. The results revealed that the nutritional structure of oats was changed by SSF, and the Avns content significantly increased, with 3.2 times more Avns in SSF oats than in unfermented oats, including 3.05, 3.09, 3.09, and 3.7 times more Avn A, Avn B, Avn C, and Avn D, respectively, and the eGI was reduced from 40.22 to 39.97 with increasing fermentation time. In general, SSF with *Monascus purpureus* has improved nutritional value, significantly increased the content of active ingredients, and reduced the level of eGI and two antinutritional factors (phytate and oxalate), which provides an effective way to improve the nutritional and digestive characteristics of oats.

## 1. Introduction

Oats are recognized as nutrient-rich and low-glycemic-index (usually referring to a GI ≤ 55) whole grains [1]. Oats have a high content and unique composition of protein, with adequate levels of essential amino acids and high levels of lysine and threonine [2,3]. Compared with other grains, oats have the highest fat content and a greater proportion of unsaturated fatty acids [4]. Moreover, the dietary fiber (DF) contained in oats, including β-glucan, arabinoxylan, and cellulose, is an important component of a healthy diet [3]. In addition, oats contain various phytochemicals, such as avenanthramides (Avns) and gamma-aminobutyric acid (GABA) [5]. Studies have indicated that these bioactive ingredients play important roles in lowering cholesterol, regulating the intestinal flora [6], and preventing atherosclerosis [7], inflammation, tumorigenesis, and antioxidation [8]. As a unique class of phenolic substances in oats [9], Avns have a low natural content and vary greatly depending on factors such as cultivation area and variety type [10]. Avn A, Avn B, and Avn C are three highly abundant components in oats [9]. However, despite the high nutritional value of oats, antinutritional factors in oat kernels can inhibit the overall absorption of nutrients and lead to reduced nutritional value [11]. Phytates and oxalates are antinutritional factors found at high levels in oats and strongly bind minerals such as iron, magnesium, calcium, and zinc, inhibiting the body’s absorption of minerals [12]. Moreover, phytates can bind amino derivatives of proteins to form insoluble complexes, reducing the bioavailability of proteins [13]. Oxalates deposit in kidney tissue or crystallize in the urinary tract, triggering kidney stones [14]. In addition to nutrients such as protein, lipids, and dietary fiber, the GI is one of the most important references for measuring the nutritional value of oats, which is especially crucial for blood glucose management [15]. It reflects the speed and ability of carbohydrates in food to raise blood glucose [16]. Low-GI foods are digested and absorbed more slowly in the gastrointestinal tract, releasing energy at a lower rate and avoiding rapid fluctuations in blood glucose, which is important for reducing the risk of type II diabetes and improving blood glucose management [17,18]. In recent years, in vitro digestive systems have become increasingly widely used in food nutrition or pharmaceutical research because they offer the advantages of being faster, more reproducible, less costly, less labor-intensive, and without ethical restriction [19]. By simulating the physicochemical environment through in vitro digestion, the eGI values of foods can be predicted simply and quickly [18].

The processing of oats subjected to heat, superheated steam, extrusion sprouting, fermentation, or supercritical fluid extraction can alter their functional and nutritional qualities to produce products with additional health benefits [3]. Fermentation is recognized as a cost-effective and green bioconversion method for improving the quality of whole-grain products [20], which can improve the bioavailability and digestibility of nutrients, enrich bioactive components, and reduce antinutritional factors [21,22]. For example, tempeh prepared with various fungi increases GABA, total phenolic content (TPC), and flavonoids in oats while reducing phytates [23]. Fermentation of oats with *Aspergillus oryzae* increases GABA levels and reduces the phytate content to varying degrees [24]. Similarly, yeast fermentation significantly increases dietary fiber content [25]. However, fermentation with lactic acid bacteria (LAB) can also reduce certain components, such as β-glucans [26,27].

*M. purpureus* is a versatile fungus that not only increases bioactive compounds such as polyphenols and flavonoids [28,29], but also produces secondary metabolites such as monacolin K and pigments, which have biological properties such as antioxidant and antimicrobial activity [30,31]. After SSF with *M. purpureus*, the L-carnitine and total flavonoid content (TFC) in sorghum increased by 1.39 and 240 times, respectively, whereas the tannin content decreased by 2.26 times and gallic and vanillic acids were newly detected. The content of certain phenolic acids, including caffeic acid, ferulic acid, vanillin, and protocatechuic acid, in fermented sorghums was 2.73–14.42 times greater than that in unfermented sorghums [28]. The carbohydrate and total protein contents of finger millet decreased after *M. purpureus* fermentation, and the reducing sugar and soluble protein contents increased. Moreover, the content of antinutritional factors was reduced, and the bioavailability of minerals was increased [29]. However, limited research has been conducted on the impact of *M. purpureus* on the nutritional structure of oats. Thus, this study evaluated changes in nutrient composition, bioactive compounds, and antinutritional factors in oats during *M. purpureus* SSF and studied their eGI by simulating in vitro digestion.

## 2. Materials and Methods

### 2.1. Materials and Reagents

Analytically pure N-hexane (M042751), petroleum ether (M045559), anhydrous ethanol (M042753), and chromatographic-grade acetonitrile were obtained from Mreda (Beijing, China). Chromatographic-grade methanol (M813904) and glacial acetic acid (A801297) were obtained from Macklin (Shanghai, China). Analytically pure hydrochloric acid (7647-01-0), sulfuric acid (7664-93-9), and acetone (67-64-1) were purchased from Sinopharm (Beijing, China). α-Amylase (S10003), amyloglucosidase (S100017), proteinase (S10051), α-amylase (S31302), pepsin (S10027), lipase (S10035), and standards of oxalic acid (B20132) and β-glucan (B21882) were purchased from Shanghai Yuanye Bio-Technology Co., Ltd. (Shanghai, China). AB-8 macroporous resin (M0042), a plant total phenol content assay kit (BC1340), and GABA content assay kit (BC6285) were obtained from Solarbio (Beijing, China). The standards of Avn A (SY1020190109018), Avn B (SY1020190109021), Avn C (SY1020190109023), and Avn D (SY202106019001981) were purchased from Shanghai Synchem Technology Co., Ltd. (Shanghai, China). A standard of oxalic acid (T2971) was from TargetMol Chemicals Inc. (Boston, MA, USA). Oat was harvested in Heilongjiang Province, China.

### 2.2. Solid-State Fermentation

The SSF process uses methods from those described by Chen et al. [32] and Wu et al. [33]. The *M. purpureus* wild-type strain (M1) is a mutant of high-yielding monacolin K and low-yielding citrinin preserved by the Beijing Food Additive Engineering Technology Research Center [30,34]. The M1 was inoculated on potato dextrose agar (PDA) (02-023) from AOBOX (Beijing, China) and incubated at 28 °C. After 3 generations of incubation, M1 spores were collected with sterile water to prepare a spore suspension (1 × 10^6^ spores/mL).

Oats were soaked in distilled water for 6 h and sterilized at 121 °C for 20 min via a vertical pressure steam sterilizer (YXQ-LS-50A) from Zhejiangxinfeng Medical Apparatus Co., Ltd. (Shangyu City, China). The oats were cooled to room temperature and then inoculated with a 10% (*v*/*w*) seed mixture. Fermentation was conducted at 28 °C for 3, 7, 14, and 21 days in a biochemical incubator (MEYZIEU-FRANCE) from Froilabo (Paris, France). The appropriate supplementation of sterile deionized water is beneficial for the growth of M1 mycelia during the SSF process [35].

After SSF, the samples were freeze-dried via a freeze-dryer (FreeZone 12plus) from Labconco (Kansas City, MO, USA) and pulverized via a mill (800C) from Shanghai Jiupin Industrial Co., Ltd. (Shanghai, China). The oat flour was sieved through a 60-mesh sieve and stored at 4 °C until further analysis.

### 2.3. Total Carbohydrate Detection

The method published by Alemayehu et al. [11] was used. The total carbohydrate content was determined via subtraction as expressed in Equation (1): the protein, fat, ash, fiber, and moisture proportions were subtracted from 100.

Total carbohydrate content (%) = [100 − (Crude protein + Moisture + Ash + Crude fat + Crude fiber)] × 100%. (1)

### 2.4. Reducing Sugar Detection

The reducing sugars were determined via the DNS method according to the method described by Miller et al. [36], with modifications. A total of 0.5 g of sample was weighed, 15 mL of water was added, a thermostat (DC-0510) from Bilon (Shanghai, China) was used in a 50 °C water bath, and the mixture was stirred continuously for 30 min on a magnetic stirrer (JK-MSH-Pro-6A) from Zhengzhou Keda Machinery and Instrument Equipment Co., Ltd. (Zhengzhou, China). The mixture was subsequently centrifuged at 4000× *g* for 5 min via a high-speed refrigerated centrifuge (3K15) from Sigma (Taufkirchen, Germany), and the supernatant was collected. The above extraction was carried out twice, and the supernatant was pooled and fixed at 100 mL for the reducing sugar to be measured. Glucose was used as a standard, and its absorbance at 540 nm was measured via an Agilent UV−Vis instrument (G9821A) (Santa Clara, CA, USA).

### 2.5. Crude Fat Detection

The methods described by Memon et al. [37] and the official method of AOAC [38] were used and modified (the extraction solvent of hexane was replaced with anhydrous petroleum ether), and the crude fat content was determined via the cable extraction method. Anhydrous petroleum ether was continuously refluxed for 6–10 h. At the end of the extraction, one drop of the extract was removed with a frosted glass rod, and the lack of oil spots on the rod indicated that the extraction was complete.

### 2.6. Crude Protein Detection

The methods of Memon et al. [37] and the official method of AOAC [38] were used. The nitrogen in the samples was estimated via a fully automated Kjeldahl nitrogen analyzer (Kjeltec™ 8200) from FOSS (Hilleroed, Denmark), and the nitrogen value was multiplied by a factor of 6.25 to determine the total protein content.

### 2.7. Dietary Fiber Detection

In accordance with the methods described by Chen et al. [39] and Ferjančič et al. [40], and official methods of AOAC [34], with modifications, 10 g of oat flour was weighed, dehydrated via the reduced pressure drying method, and treated with petroleum ether and 85% ethanol solution for defatting and deglycosylation, respectively, and the weight change was recorded to calculate the factor of change in mass of the treated samples (f).

Samples of 0.25–3 g were weighed, placed in 35 mL of 50 mmol/L maleic acid buffer, and enzymatically digested with α-amylase (S10003), amyloglucosidase (S100017), and proteinase (S10051) for the determination of total dietary fiber (TDF), insoluble dietary fiber (IDF), and soluble dietary fiber (SDF), respectively. The enzymatic conditions are shown in Table 1.

### 2.8. β-Glucan Detection

After modification according to the methods of Heidary Vinche et al. and Harasym et al. [41,42], 2 g of oat flour was weighed, and 75% anhydrous ethanol was added at a mass-volume ratio of 1:8. The mixture was extracted at 80 °C for 2 h to remove fat-soluble substances, free sugars, and small-molecule proteins, inactivating the endogenous β-glucanase. Then, it was centrifuged at 8000 rpm for 10 min by a high-speed refrigerated centrifuge, and the supernatant was discarded. The precipitates were subsequently dried at 60 °C. The treated samples were added to distilled water at a mass-volume ratio of 1:20, the mixture was heated at 65 °C for 30 min, high-temperature-resistant α-amylase was added to remove starch (the amount of enzyme was approximately 1/200 of the total amount of material-liquid), and starch was detected to determine whether it had been completely removed with a potassium iodide solution of starch. After the starch was removed, the pH was adjusted to 9, and extraction continued for 1 h. The proteins were removed from the supernatant via the isoelectric point method (the pH of the supernatant was adjusted to 4.5 with 6 M HCl, and the mixture was allowed to stand for 30 min at 95 °C in a water bath). The supernatant was collected by centrifugation at 10,000 rpm for 10 min, and subsequently precipitated with ethanol (final ethanol volume fraction of 60%) at 4 °C for 10 h. Finally, the precipitate was obtained by centrifugation at 8000 rpm for 20 min. The precipitate was redissolved, and the volume was fixed to 10 mL, which was the β-glucan solution to be measured. β-glucan was used as the standard. A small amount of deionized water was added to a 70 °C water bath to facilitate solubilization, and the mixture was allowed to cool to room temperature, after which the volume was increased. First, 0, 0.02, 0.04, 0.06, 0.08, 0.1, or 0.14 mL of the standard solution was mixed with deionized water to make a volume of 2 mL; 4 mL of Congo red solution was added; and the mixture was allowed to react for 30 min. The absorbance at 545 nm was measured via UV−Vis (G9821A). Then, 10 mg of Congo red was dissolved in 0.1 mol/L pH 8.0 phosphate buffer, and the volume was 100 mL.

### 2.9. Ash Content Detection

In accordance with the method described by the official method of AOAC [38], with modifications, the weight of a clean and dry crucible (m1) was measured, and 5 g of sample (m2) was added and carbonized in an electric heating jacket. After carbonization, the samples were placed in a muffle furnace (LE14/11/B150) from Nabertherm GmbH (Lilienthal, Germany) and burned at 550 °C for 5 h until the samples turned white or gray. The crucible was cooled in a desiccator and weighed (m3). The ash content was determined via Equation (2).

Ash (%) = (m2 − m1)/(m3 − m1) × 100. (2)

### 2.10. Total Phenol Detection

The methods published by Bai et al. and Sun et al. were used [43,44]. A total of 1.5 g of oat flour was weighed and defatted with n-hexane, and 60% anhydrous ethanol was added at a mass-volume (g/v) ratio of 1:25. The mixture was ultrasonically extracted for 10 min at a power of 90 W via an ultrasonic extractor (BILON-1000CT) from Bilon (Shanghai, China), stirred continuously for 1 h on a magnetic stirrer in a water bath at 60 °C, and ultimately centrifuged at 7000 rpm for 15 min in a high-speed refrigerated centrifuge. The supernatant was preserved, and the extraction was repeated three times. The supernatant was evaporated to a volume of 100 mL via a rotary evaporation system (R-300) from BUCHI (Flawil, Switzerland). Gallic acid was used as a standard. A plant total phenol content assay kit (BC1340) was used, and the absorbance at 760 nm was measured via UV−Vis.

### 2.11. Avenanthramide Detection

In accordance with the methods of Jágr et al. and Lee et al., with modifications [45,46], 5 g of oat flour was weighed and defatted with n-hexane, and 80% anhydrous ethanol was added at a mass-volume ratio of 1:20. The mixture was ultrasonically extracted for 10 min at a power of 90 W, stirred continuously for 2 h on a magnetic stirrer in a 50 °C water bath, and ultimately centrifuged at 8000 rpm for 10 min in a high-speed refrigerated centrifuge. The supernatant was preserved. The extraction was repeated three times. The supernatant was decontaminated with AB-8 macroporous resin (M0042), which was washed with 3-column volumes of purified water to remove the water-soluble pigments, and the 6-column volumes of 95% the ethanol washings were collected. The ethanol mixture was evaporated to a volume of 5 mL via a rotary evaporation system and then redissolved in chromatographic grade methanol and fixed in volume. Samples are diluted as needed for testing. Avn A (SY1020190109018), Avn B (SY1020190109021), Avn C (SY1020190109023), and Avn D (SY202106019001981) standards were dissolved in methanol.

Avns were analyzed via high-performance liquid chromatography (HPLC). Avns were quantified using a coupled HPLC system (LC-20A) (Shimadzu, Kyoto, Japan) and a C18 column (5020–03346) (Shimadzu, Kyoto, Japan). The following parameters were used: C18 column 150 nm × 30 nm, 5 μm, mobile-phase flow rate, 0.8 mL/min; detection wavelength, 340 nm; and detection temperature, 30 °C. The mobile-phase conditions are shown in Table 2.

### 2.12. γ-Aminobutyric Acid Detection

After modification according to the methods of Al-Ansi et al. and Cai et al. [23,47], oat flour was defatted with n-hexane, added to ultrapure water at a mass-volume ratio of 1:20, ultrasonicated at 90 W for 10 min, and extracted in a water bath at 55 °C for 2 h. The assay was performed via a GABA content assay kit (BC6285).

### 2.13. Detection of Antinutritional Factors

In accordance with the modified methods published by Abbou et al. and Alemayehu et al. [11,48], the antinutritional factors were detected as follows:

Phytate analysis: 0.1 g of sample was mechanically shaken in 10 mL of 2.4% HCl aqueous solution at room temperature for 1 h and centrifuged at 10,000 rpm for 15 min in a high-speed refrigerated centrifuge, and the supernatant was retained and fixed to 10 mL. Two milliliters of a water solution with 0.3% sulfosalicylic acid and 0.03% FeCl_3_(H_2_O)_6_ was added to 3 mL of the supernatant and mixed thoroughly for 5 s. Phytic acid standards were prepared in 0.2 N HCl solution, and the absorbances of the standards and samples were measured at 500 nm using UV−Vis.

Oxalate analysis: A total of 40 mL of 2.0 M HCl was used to extract 1 g of sample, and the samples were stirred constantly for 3 h at room temperature and centrifuged at 8000 rpm for 30 min in a high-speed refrigerated centrifuge. Then, the supernatant was retained and fixed to 50 mL. Eight milliliters of a water solution with 0.3% sulfosalicylic acid and 0.03% FeCl_3_(H_2_O)_6_ was added to 3 mL of the supernatant, which was mixed thoroughly for 5 s. The oxalic acid standard was dissolved in a 2.0 M HCl solution, and the absorbances of the standards and samples were measured at 510 nm using UV−Vis.

### 2.14. In Vitro Digestion

In vitro digestion methods refer to standard in vitro digestion simulation methods [49]. The simulated saliva fluid, simulated gastric fluid (SGF), and simulated intestinal fluid (SIF) electrolyte stock solutions were prepared according to the method of an enzyme solution created in electrolyte stock solution. For the oral digestion stage, 1 g of the sample was mixed with 5 mL of water at 100 °C for 30 min and cooled to 37 °C, after which 3.5 mL of simulated saliva fluid electrolyte stock solution and 0.5 mL of α-amylase (S31302) solution was added. The mixture was supplemented with 25 μL 0.3 M CaCl_2_ and 975 μL of water, and the concentration of α-amylase in the final mixture was 75 U/mL. For the gastric digestion stage, the pH of the oral digested sample was adjusted to 3.0 with 1M HCl, 9.1 mL of a mixed solution of SGF electrolyte stock solution and pepsin (S10027) was added, 5 μL 0.3 M CaCl_2_ and water were added, and the concentration of pepsin in the final mixture was 2000 U/mL. For intestinal digestion stage, the pH of the gastric digestion sample was adjusted to 7.0, with 1 M NaOH, 11 mL of SIF electrolyte stock solution, and 5 mL entero-digestive enzymes (consisting of α-amylase (S31302), trypsin (S10033), and lipase (S10035)), and 2.5 mL of bile salts and 40 μL 0.3 M CaCl_2_ and water were added. The volume of water during the gastric and enteric digestion stages will vary depending on the volume of HCl and NaOH added.

Oral and gastric digestion was maintained for 2 min and 30 min, respectively. Starting from the intestinal digestion stage, 1 mL of digest was aspirated at 0, 5, 10, 30, 60, 90, 120, 150, and 180 min; the enzyme was inactivated at 100 °C; and the glucose content was detected via the DNS method. The eGI is calculated via Equation (3) [18]. Hydrolysis index (HI): The area under the hydrolysis rate curve of the sample versus the area under the hydrolysis rate curve of the control sample (white bread).

eGI = 39.71 + 0.549 HI(3)

### 2.15. Statistical Analysis

Statistical processing of the data was carried out via SPSS 17.0 software (IBM, New York, NY, USA), and the data are presented as the means ± standard deviations. All the data were analyzed via one-way ANOVA supplemented with Duncan’s multiple-range test, and significant differences between groups were considered to be significant at *p* ≤ 0.05. The data were visualized with OriginPro 2024 (OriginLab, Northampton, MA, USA). The experiments were repeated three times for each measurement.

## 3. Results

### 3.1. Status of Oats at Different Time Points After SSF with M. purpureus

**Figure 1:**
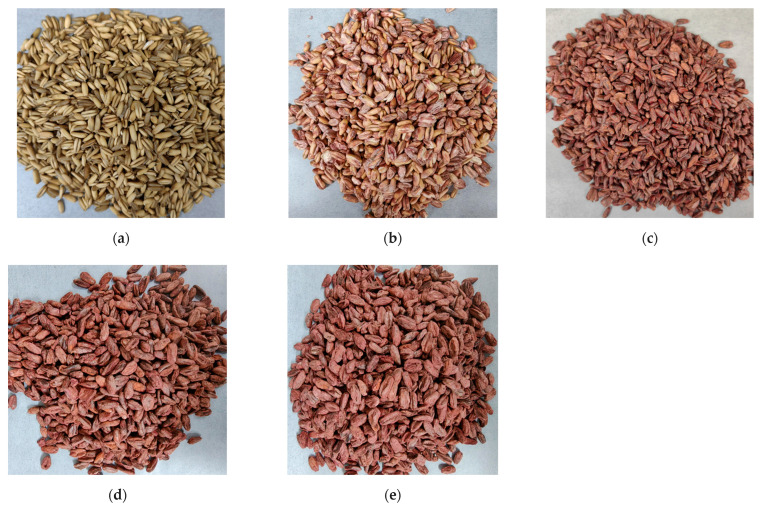
The state of the oats after SSF with *M. purpureus*. (**a**) 0 days; (**b**) 3 days; (**c**) 7 days; (**d**) 14 days; (**e**) 21 days.

As shown in Figure 1, the state of the oats after SSF with *M. purpureus* for 0, 3, 7, 14, and 21 days was altered. Owing to the color changes, mycelia and pigmentation were evident on the surface of the oats on the third day of SSF, and the surface of the oats was essentially covered by *M. purpureus* on the seventh day of SSF. During the processing of freeze-dried oats, it was found that oats with 21 days of SSF were more prone to breakage.

### 3.2. Alterations in the Nutrient Composition of Oats upon SSF

**Figure 2:**
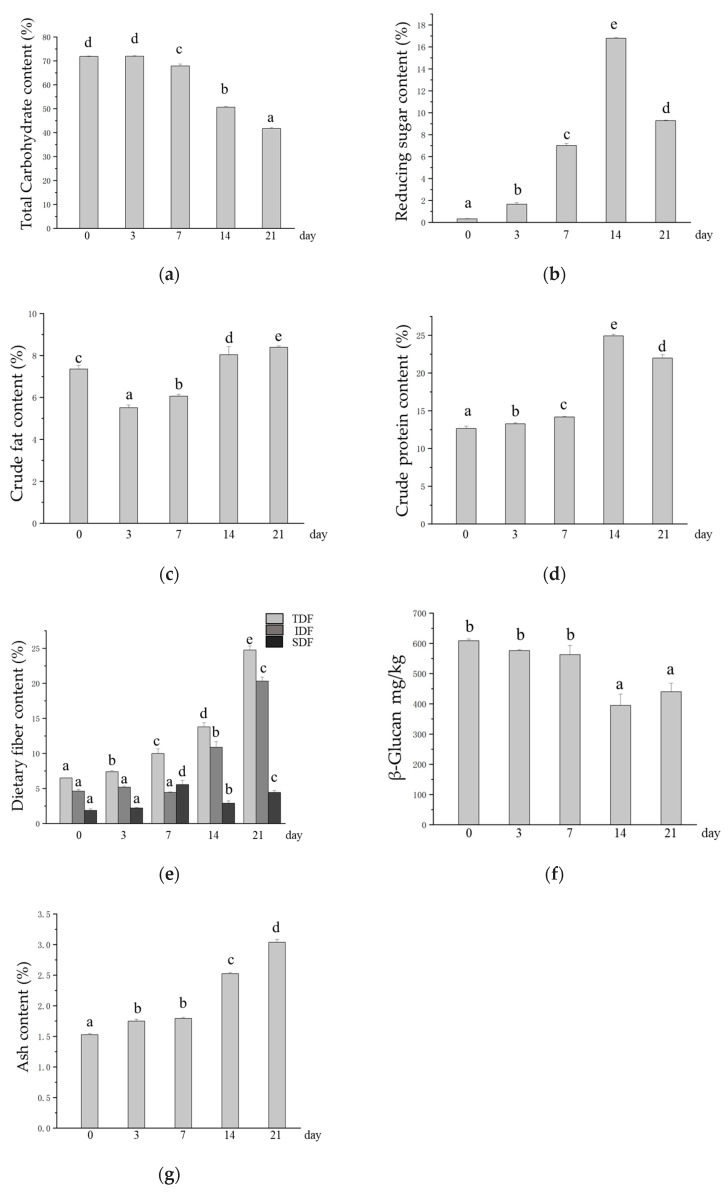
Changes in the contents of base nutrients in oats after SSF. (**a**) Changes in total carbohydrate content; (**b**) changes in the content of reducing sugars; (**c**) changes in crude fat content; (**d**) changes in crude protein content; (**e**) changes in dietary fiber content, TDF: total dietary fiber, IDF: insoluble dietary fiber, SDF: soluble dietary fiber; (**f**) changes in the content of β-glucan; (**g**) changes in ash content. Different lowercase letters indicate significant differences among groups (*p* ≤ 0.05).

*M. purpureus* uses starch as a carbon source for metabolite growth and synthesis during fermentation [20], and as a result, a significant decrease in the total carbohydrate content in oats after SSF was observed with increasing fermentation time (Figure 2a). This may enhance glucose homeostasis, improve the control of blood glucose levels, and reduce the risk of glucose-related complications [20,50]. Moreover, a reduction in total carbohydrates means an increase in the relative amount of other nutrients, such as protein and dietary fiber. This enables oats to provide the body with a richer supply of nutrients while providing a sense of satiety. The reducing sugar content in oats after SSF increased significantly from 3 to 14 days of SSF, with the highest content of 16.7% occurring at 14 days of SSF (Figure 2b). The crude fat content was significantly lower than that in unfermented oats at 3 and 7 days of SSF but was again elevated in SSF oats at 14 and 21 days (Figure 2c). The crude protein content significantly increased in oats after SSF, reaching 12.64% in unfermented oats and 24.91% in SSF oats after 14 days (Figure 2d). Fungal mycelial proteins produced by microorganisms during growth are the main reason for the increased crude protein content in SSF oats [24]. The TDF content continued to increase during SSF, the IDF increased significantly in the later stages of SSF, and the SDF content reached its highest value at 7 days of SSF. The TDF, IDF, and SDF contents in unfermented oats were 6.50%, 4.63%, and 1.87%, respectively, and the highest levels of TDF, IDF, and SDF reached 24.75%, 20.31%, and 5.55%, respectively, after SSF (Figure 2e). A relative increase in dietary fiber content helps increase the number and diversity of beneficial bacteria, enhance the structure and function of the intestinal microbiota, maintain normal intestinal digestion and absorption functions, and prevent intestinal diseases such as constipation and diarrhea [51,52]. β-glucan is an SDF with multiple health benefits, such as maintaining blood glucose levels, lowering cholesterol, and regulating the intestinal flora [6]. However, in this study, there was no significant difference in the content of β-glucan after SSF with *M. purpureus* at 0, 3, or 7 days; the content was significantly reduced after 14 and 21 days of SSF; and there was no significant difference between 14 and 21 days of SSF (Figure 2f). The ash content can reflect the total mineral content of the grain to some extent. The ash content in SSF oats was significantly greater than that in unfermented oats, ranging from 1.53% in unfermented oats to 3.04% in SSF oats (Figure 2g).

### 3.3. Change in the Active Ingredient Content in Oats After SSF

**Figure 3:**
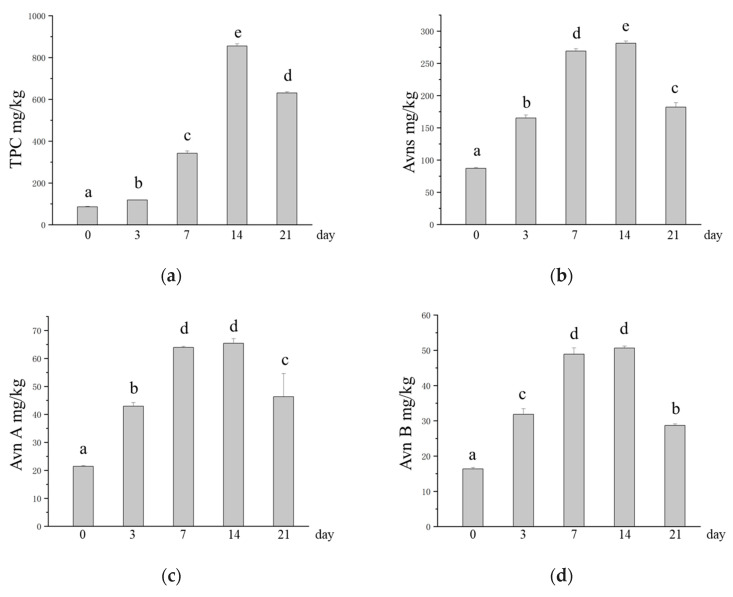

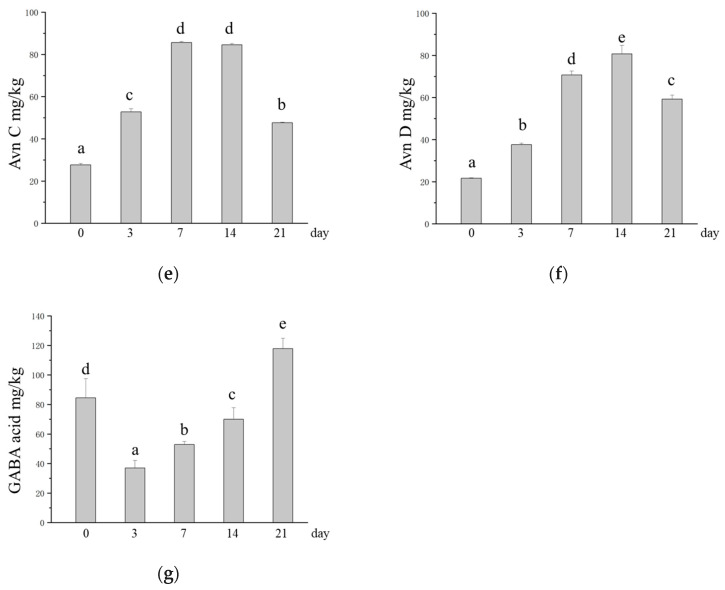
Changes in the contents of phytochemicals in oats after SSF. (**a**) Change in TPC; (**b**) changes in the content of Avns; (**c**) change in the content of Avn A; (**d**) changes in the content of Avn B; (**e**) changes in the content of Avn C; (**f**) changes in the content of Avn D; (**g**) changes in the content of GABA. Different lowercase letters indicate significant differences among groups (*p* ≤ 0.05).

Studies have shown that microbial SSFs can release active compounds under suitable conditions [20]. During fermentation, *M. purpureus* can disrupt the binding of bound active components to structural components of the oat cell wall (cellulose, pectin, hemicellulose, lignin, etc.) to release and produce functional metabolites [53,54]. The TPC increased from 85.83 mg/kg to 855.42 mg/kg after SSF with *M. purpureus*, which was 9.97 times greater in SSF oats than in unfermented oats (Figure 3a). Avns are phytochemicals that are unique to oats, and their potential health benefits, such as reducing cancer cell growth and migration and inhibiting atherosclerosis, are gradually being discovered [7,55]. The experimental results revealed that the content of Avns significantly increased after *M. purpureus* SSF, and the Avn (A + B + C + D) content increased from 87.19 mg/kg to 281.32 mg/kg (Figure 3b). The contents of Avn A, Avn B, Avn C, and Avn D in unfermented oats were 21.43 mg/kg, 16.37 mg/kg, 27.69 mg/kg, and 21.70 mg/kg, respectively, and the contents of Avn A, Avn B, Avn C, and Avn D in SSF oats were the highest at 65.40 mg/kg, 50.64 mg/kg, 84.58 mg/kg, and 80.70 mg/kg, respectively, approximately three times greater (Figure 3c–f). The Avns and phenolic compounds contained in oats have been shown to have good antioxidant properties [9,56]. After fermentation, the content of phenolic compounds increases along with their active function [24]. GABA is a nonprotein amino acid that is the main inhibitory neurotransmitter in the central nervous system and lowers blood pressure, which has a positive impact on human health [57]. However, in this study, we found that the GABA content after *M. purpureus* SSF was lower than that of unfermented oats at 3, 7, and 14 days of fermentation, with the lowest levels occurring at 3 days of SSF (Figure 3g).

### 3.4. Effects of SSF on the Contents of Antinutritional Factors in Oats

**Figure 4:**
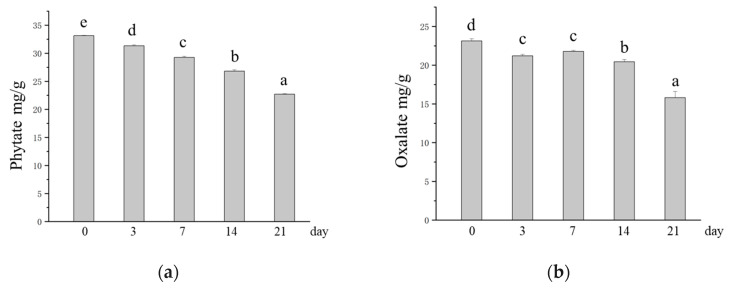
Changes in the contents of antinutritional factors in oats after SSF. (**a**) Changes in phytate content; (**b**) changes in oxalate content. Different lowercase letters indicate significant differences among groups (*p* ≤ 0.05).

Phytates and oxalates can have deleterious effects on human nutrition and health. It has been widely reported that there are no enzymes in the human body that degrade phytates in the gastrointestinal tract. As a result, phytate-rich diets result in dietary minerals not being adequately absorbed by the body [23]. Oxalates are among the main factors contributing to the development of hyperuricosuria, which can cause calcium oxalate deposits and lead to kidney stones [58]. Therefore, the reduction in phytate and oxalate contents in oats caused by *M. purpureus* SSF may have a positive effect on consumers. Compared with those in unfermented oats, the phytate and oxalate contents in SSF oats decreased significantly with increasing SSF time. The phytate content decreased from 33.13 mg/g to 22.69 mg/g, and the oxalate content decreased from 23.15 mg/g to 15.82 mg/g (Figure 4a,b). SSF is recognized as an important means of reducing phytic and oxalate in grains, which is achieved through the degradation of phytases and decarboxylases produced by microorganisms [59]. This is supported by previous studies. Shi et al. [60] reported that phytate content was greatly reduced by SSF of whole wheat with *Monascus* sp.. Ojha et al. [61] reported that fermentation of sorghum flour by Lactobacillus plantarum significantly reduced the phytate and oxalate contents in the substrate by 77% and 67.85%, respectively. The antinutritional factor content is reduced by SSF, which releases more minerals, thus increasing the bioavailability of minerals in oats [24].

### 3.5. Influence of SSF on the Digestibility of Oat

The GI is a measure of the postprandial glycemic response induced by food intake, reflecting the rate and ability of a food to increase blood glucose compared with glucose [62]. Therefore, an in vitro digestion assay of the GI values of fermented oats can potentially reveal the level of blood glucose induced by SSF oats during digestion. As shown in Table 3 and Table 4, the hydrolysis rate, hydrolysis index (HI) and eGI of oats were decreased with continuing SSF. This suggests that the SSF influenced the digestibility of oat.

## 4. Discussion

Oats have recently gained popularity because of their high nutritional value and health benefits. The processing of oats results in the production of more beneficial substances during processing and reduces the number of substances that have a negative effect on human health [60,63]. In this study, we found that the SSF with *M. purpureus* significantly changed the nutritional structure of oats, which is in general agreement with the findings of previous studies [29]. The contents of basic nutrients, including reducing sugars, crude protein, TDF, IDF, and ash, increased significantly, and the content of phytochemical phenolic compounds increased significantly. However, we also found that total carbohydrate and β-glucan contents were significantly reduced throughout the SSF period, crude fat content was significantly reduced at 3 and 7 days of SSF and was subsequently increased at 14 and 21 days of SSF, SDF content reached its highest value at 7 days of SSF and then significantly decreased with the extension of SSF time, and GABA content was also significantly reduced at SSF for 3–14 days. The main reason is that microbial activity utilizes starch, soluble sugars, and lipids in grains through glycolysis, lipolysis, and other pathways without adding additional carbon and nitrogen sources [64], and pigment production affects fat and GABA contents during the growth of *M. purpureus* [65]. The fungal mycelial proteins produced and the enzymes required for life activities by *M. purpureus* are the main reasons for the increased crude protein content in SSF oats. In addition, antinutritional compounds, including phytates and oxalates, in grains are hydrolyzed during SSF. Moreover, when comparing our results with those of previous studies, it is important to note that we focused on Avns and the eGI of SSF oats, which have not been investigated in previous studies of SSF. We found that the content of Avns in SSF oats significantly increased, including the contents of four isomers (Avn A, Avn B, Avn C, and Avn D), which also differentially increased. The in vitro digestion results indicated that the reduction in the β-glucan content in the SSF oats did not increase the eGI of the SSF oats. The eGI of oats after *M. purpureus* SSF decreased significantly with increasing fermentation time, so the oats remained in the low-GI range.

Carbohydrates are usually the preferred carbon source for most fungi and bacteria used in SSF. During SFF, carbohydrates are enzymatically hydrolyzed to ultimately produce glucose monomers, which are used to sustain microbial life activities [66]. Carbohydrates are the energy source for microbial cellular respiration and other metabolic activities, such as cell division and material synthesis [67,68]. In addition, *M. purpureus* fermentation consumes carbon to produce a variety of physiologically important secondary metabolites, such as red pigments and monacolin K [30,31]. Thus, SFF significantly reduces the amount of carbohydrates in the fermentation substrate and increases the amount of reducing sugars in the fermentation substrate. During SSF, the content of crude fat decreases in the early stage but increases in the late stage, which is strongly associated with the metabolic activity of microorganisms. In the early stage of *M. purpureus* infection, microorganisms adapt to the environment and grow rapidly. On the one hand, fat is broken down into intermediates such as acetyl coenzyme A through metabolic pathways such as β-oxidation and then enters the tricarboxylic acid cycle for complete oxidative decomposition. Moreover, acetyl coenzyme A is a key metabolite involved in central carbon and energy metabolism and is the most important substrate for pigment biosynthesis. On the other hand, the lipolytic secreted by *M. purpureus* breaks down the fat in oat material into glycerol and fatty acids, which provides energy for the growth and reproduction of microorganisms and raw materials for the synthesis of cellular materials, thus causing a decrease in the crude fat content [31,69]. As SSF progresses, the crude fat content increases, which can be attributed to the increased biomass of mycelia and the production of fat-soluble pigments [70]. The fat content was reduced after 2 days of fermentation of oats with *Monascus sp.* by Shi et al. [60]. The fat content was enriched after 14 days of fermentation of oats with *P. ostreatus* by Espinosa-Páez et al. [70,71]. Thus, shorter fermentation times may favor more lipolysis of intrinsic oat lipids as a carbon source for the fungus, whereas lipogenesis is more likely to be used for the development of fungal mycelia and the synthesis of pigments in longer fermentations [72]. The microorganism’s proliferation results in the production of protein enzymes to carry out the necessary microbial activities, and these enzymes themselves can increase protein content [73,74]. In fungal SSF systems, the proliferation of fungal cells increases mycelial biomass, and proteins are one of the components of the mycelium, thus further increasing protein content [75]. Simultaneous fermentation increases the digestibility of plant proteins and the soluble protein content [70,76]. During SSF, microorganisms can break the cell wall of oat cells and release lower-molecular-weight proteins from the cell wall, making the proteins easier to digest. Various enzymes secreted by microorganisms, such as proteases, can act on the proteins in the raw material and break them down into smaller peptide segments and amino acids. Peptides and amino acids have better solubilities than larger proteins do, thus increasing the amount of soluble protein in the system [68,72,77]. DFs are one of the components of the cell wall and exhibit a complex network structure [78]. The cellulase and hemicellulase secreted by microorganisms during fermentation can destroy the structure of the cell wall. On the one hand, DFs that were originally bound by the cell wall or were difficult to extract and measure internally were released; on the other hand, the cross-linking structure between some of the original DF molecules may also be destroyed, resulting in a looser structure of DFs, thus increasing the detected TDF content. Owing to the looser structure of DF, the specific surface area is increased, thus increasing water adsorption, and the increase in SDF in the SSF period may also be caused by these factors [79]. As SSF continues, microorganisms may begin to use SDF as a carbon source and energy source to maintain their growth and metabolism after consuming easily available nutrients from the carbon source as their living environment continues to change [80]. The excessive hydrolysis of β-glucan with increasing SSF time and microbial population resulted in a decrease in the amount of extracted β-glucan, which may be related to the utilization of SDF as a carbon source. Zhang et al. verified via SSF experiments in oats using *Lactobacillus plantarum* that no significant changes in soluble dietary fiber or β-glucan content were observed after 28 and 40 h of SSF, respectively [81]. Although prolonged SSF can reduce the total amount of β-glucan, filamentous fungi have been shown to increase its extractability and make it more bioavailable through hydrolysis by α-amylase and β-glucosidase biopolymers [72,82]. The increase in ash content due to SSF may be related to the loss of other nutrients, especially a decrease in carbohydrates [70]. Other reports indicate that SSF increases the content and bioavailability of certain free minerals in grains, including magnesium, iron, calcium, and zinc. Minerals present in plant products have very low bioavailability because of the formation of indigestible complexes with other substances. Fermentation releases minerals by degrading antinutritional factors (phytates and oxalates) and disrupting cellular structures [67,72].

Phenolic compounds are present in three forms, free, bound, and conjugated, and the most common phenolic compounds in oats are bound to polysaccharides and proteins in the cell wall skeleton through ester bonds. These phenolic compounds are virtually impossible to utilize further, and they must be present in the free form to confer biological activity [32,83]. A number of carbohydrate-hydrolyzing enzymes produced by microorganisms are key to increasing the content and activity of oat phenolic compounds in SSF, including α-amylase, cellulase, β-glucosidase, and xylanase, which break down the tight structure of the grain’s cell wall, allowing phenolic compounds bound to the cell wall to be released [56,84]. The *M. purpureus* SSF enhances the ability to accumulate carbohydrate hydrolases, and phenolic mobilization in SSF oats is associated with the activity of carbohydrate hydrolases produced by *M. purpureus*. Bei et al. [85] investigated the enzymatic mechanism of phenolic release by carbon hydrate hydrolase during *M. purpureus* SSF. Phenolic mobilization is closely related to the activities of α-amylase, xylanase, and cellulase, and phenolic release is a result of the enzyme breaking down the cell wall structure. The second mode of increasing the active content of phenolics is transformation by other compounds through the shikimate and malonic acid pathways [72]. SSF with *Monascus anka* and *Bacillus subtilis* has been shown to increase the phenolic content 23-fold, improve the antioxidant capacity, and modify the phenolic composition of oats [32]. In addition, fermentation enriches the phenolic constituents (gallic acid, p-hydroxybenzoic acid, caffeic acid, vanillic acid, p-coumaric acid) in oats [32,56]. Avns are phenolics that are uniquely present in oats and have multiple health benefits for humans, such as cancer prevention, antioxidant and anti-inflammatory properties, and maintenance of muscle health [9]. However, few studies have reported changes in the contents of Avns in oats after microbial fermentation. The release of bound Avns is an important reason why SSF can increase the Avn content in oats. Another reason may be that the *M. purpureus* SSF process can produce enzymes that modify Avns. Owing to their chemical structure, oat Avns are composed of amides of different hydroxycinnamic acids and different o-aminobenzoic acids. Avn A, Avn B, and Avn C are formed by the condensation of 5-hydroxy o-aminobenzoate with p-coumaric acid, ferulic acid, and caffeic acid, respectively. *M. purpureus* SSF can increase the content of soluble phenolics, but changes in the content of o-aminobenzoic acids have seldom been reported. Aves synthesized via the phenylalanine pathway or tyrosine pathway involve three enzymes, phenylalanine ammonia-lyase (PAL) or tyrosine ammonia-lyase (TAL), 4-coumarate: coenzyme A ligase (4CL), and hydroxyanthranilate N-hydroxycinnamoyltransferase (HHT) [9,86]. PAL catalyzes the formation of trans-cinnamic acid from L-phenylalanine, which, in the metabolic pathway of fungal microorganisms, can be produced through the shikimate pathway. 4CL catalyzes the generation of cinnamic acid and its hydroxyl or methoxy derivatives to produce the corresponding coenzyme A esters, as well as one of the key enzymes in the synthesis of cellulose, and HHT, a member of the BAHD acyltransferase superfamily; catalyzes the N-acylation of 5-HHT, a member of the BAHD acyltransferase superfamily; and catalyzes the N-acylation of 5-hydroxyphthalamic acid with p-coumaroyl-coenzyme A or caffeoyl-coenzyme A but not feruloyl-coenzyme A for the synthesis of Avn A and Avn C. Avn B (ferulic acid derivatives) are produced by the demethylation of Avn C [87,88]. Therefore, although no study has clearly indicated that *M. purpureus* contains key enzymes for synthesizing Avns, the *M. purpureus* SSF may increase the content of Avns in SSF oats through the modification of Avns by enzymes produced by microorganisms.

GABA is a potent bioactive substance widely found in nature and is produced by L-glutamic acid catalyzed by glutamic acid decarboxylase (GAD), which has a variety of physiological functions, such as enhancing immunity, relieving anxiety, and regulating blood pressure [89,90]. To date, fungi and bacteria have been shown to be able to synthesize GABA [91]. The culture conditions of microorganisms significantly affect the GABA production capacity, carbon and nitrogen sources, and pH and GAD activity. These are important parameters for GABA production, and pH is a key factor affecting GAD activity [92,93]. In our study, we found that the GABA content in *M. purpureus* SSF oats decreased in the early stage but increased in the late stage without additional carbon and nitrogen sources, which might be affected by the growth of *M. purpureus* and related enzyme activities [94]. In the early stage of fermentation, *M. purpureus* begins to grow and multiply in oat media, where it performs several basic metabolic pathways to establish its own growth and metabolic basis, and it needs to utilize a variety of nutrients, such as carbon and nitrogen sources, in the oats to satisfy its own growth requirements. In this process, nutrients are used to synthesize microbial cell structures and other metabolites [89]. Second, the activity of GAD involved in GABA synthesis may be relatively low due to environmental factors (pH, temperature, etc.), and the rate of GABA synthesis cannot compensate for the rate of utilization of *M. purpureus*; thus, the GABA content appears to be temporarily reduced. With continuing fermentation, some acidic substances are produced in the metabolic process of *M. purpureus*, which changes the environment of the SSF system, reduces the pH value, and activates the enzyme activity of GAD, and the microbial GABA synthesis pathway is strengthened. Moreover, amino acids such as glutamic acid produced by protein degradation provide sufficient raw materials for the synthesis of GABA, thus promoting the accumulation of GABA. Many studies have reported that LAB is considered the most technologically relevant and competitive microbiome for synthesizing GABA compared with other strains of microorganisms, as LAB acts as a probiotic, producing high levels of biologically active compounds and postbiotics that have a positive impact on health [92,95].

The main antinutritional factor present in oats is phytate, which hinders the absorption of minerals in the human body. Microorganisms secrete phytases and decarboxylases during fermentation, which hydrolyze phytic acid (to inositol and phosphate) and degrade oxalate (to CO₂ and formate), respectively [61,96]. The use of microorganisms such as *Lactobacillus plantarum*, yeast, and *Aspergillus oryzae* reduces the phytate and oxalate contents of grains to varying degrees. Tosun et al. [76] investigated the effects of a number of different microbial fermentations on oat phytates and reported that phytates decreased in all fermentation systems, with LAB fermentation resulting in the lowest phytate degradation and yeast fermentation resulting in the highest phytate degradation. Jagannath et al. [96] reported that *Lactobacillus* strain fermentation of green vegetables with antinutritional factors also had a degrading effect.

In in vitro digestion experiments, grains can respond to the degree of hydrolysis of the grains and thus indirectly respond to the rate of glucose release from the grains during digestion. SSF reduces eGI levels, which is directly related to the reduction in carbohydrates in the oat matrix after SSF. Fermentation can alter starch composition and digestibility, and the digestibility of grain starch, especially resistant starch (RS), is highly correlated with the glycemic response [68,77]. Adebo et al. [97] reported that grain RS prolongs the release of glucose during digestion, thereby controlling the rate of blood glucose increase. Gong et al. [77] reported that *Lactobacillus*-fermented purple potato flour increased the RS content, decreased the content of rapidly digestible starch and slowly digestible starch, and reduced the HI and predicted glycemic index. In vitro digestion assays are a common means of rapidly determining the digestibility of nutrients in foods and are a useful method for categorizing foods on the basis of their postprandial glycemic response. In vitro GI assays are simple, rapid, and require minimal skill and infrastructure, allowing multiple food samples to be tested simultaneously [98].

Enzymes produced by microbial metabolic activities during fermentation can change the ratio of various nutrients and antinutritional factors and improve nutrient digestibility and bioavailability [21,99]. The degree of influence on the nutritional structure of grains varies depending on the fermentation strains used [100]. For example, fermentation of seven different grains with *Sanghuangporus sanghuang* altered their nutrient composition by consuming fats and starches and converting them into soluble proteins and sugars. A significant positive correlation was observed between the mycelial growth rate and the starch content of the grains [101]. Similarly, fermenting corn with yeast and bacteria increases its crude protein, crude fat, and crude fiber contents while significantly reducing its ash content and antinutritional factors [102]. Moreover, many studies have shown that fermentation not only enriches the phytochemicals in grains but also enhances their active functions, such as phenolic acids, flavonoids, and alkaloids [21]. Chen et al. [32] showed that the total phenols in oats fermented by *Monascus anka* and *Bacillus subtilis* presented high antioxidant capacity by assaying the scavenging effects of DPPH and ABTS. Fermentation of grains by LAB isolated from fermented beverages resulted in significant increases in TPC, TFC, GABA, and glucuronic acid contents, and the DPPH and ABST results indicated a significant increase in free radical scavenging capacity, as well as a significant increase in the inhibitory effect on angiotensin transferases [99]. The SSF of *Xylaria nigripes* (XN) significantly increased the TPC and TFC of whole grains and exhibited greater antioxidant, anti-inflammatory, and neuroprotective potential. Compared with unfermented grains, all XN-fermented grains presented greater DPPH free radical scavenging activity and reducing power and significantly better inhibition of lipid peroxidation and NO [103].

In SSF, each fermentation experiment has a different pathway of metabolic action on the same substrate subjected to fermentation conditions, and the order and extent of substrate utilization by the microorganisms, as well as the products of synthesis, can vary greatly. Microorganisms largely determine the composition and function of the final fermented grains [100]. Differences in fermentation environments, such as temperature, moisture content, and pH, also play crucial roles in the SFF process [97]. The grain substrate provides carbon and nitrogen sources for microbial growth, whereas microbes provide the enzymes needed for metabolic activity to determine which biochemical pathways are used for microbial growth and proliferation, and environmental factors help to regulate the optimal fermentation conditions to direct fermentation to produce the desired compounds and products [72,104].

SSF is now a viable and efficient processing technology for the food industry, with significant advantages in the development of functional foods, improvement in product flavors, enhancement in product processing characteristics, and enhancement in nutrient content [21,105]. Study by Lu et al. [27] on *Lactobacillus plantarum*-fermented oat sourdough revealed that SSF retained higher levels of soluble β-glucan (2.18%), high molecular weight fractions, and viscosity, which were beneficial for the improvement in texture and health benefits (delayed glucose uptake) of gluten-free bread. The highland barley was treated to significantly enhance its nutritional value and processing characteristics through solid-state fermentation by *Lactobacillus acidophilus* (LAC), and the biscuits made from LAC-fermented highland barley flour had significantly higher protein (8.92 g/100 g) and total dietary fiber (13.03 g/100 g) contents than commercially available products [106].

## 5. Conclusions

This study revealed that *M. purpureus* SSF could affect the contents of various substances in oats to different degrees and change the nutritional structure of oats. On the basis of the experimental results, oats with an SSF of 7 days or 14 days are considered to be more suitable for processing and consumption. Compared with unfermented oats, SSF oats contained significantly lower carbohydrate contents; higher crude protein, dietary fiber, and ash contents; higher levels of the phytochemicals Avn and TPC; and lower levels of the antinutritional factors phytate and oxalate. Although Avns, as active phytochemicals, have a significant effect on the prevention of chronic diseases [5], their natural level is low, and the present study of fermented oats with the study of Avns in fermented oats can provide a source for their development into food products that are beneficial to human health. We will subsequently continue to focus on changes in the activity of phytochemicals in fermented oats to provide more possibilities for the application of oats.

## Figures and Tables

**Table 1:** The enzymatic conditions *.

Name of the Enzyme	Concentration	Volumetric	Temperature	pH	Time
α-Amylase	800 U/mL	5 mL	60 °C	6.0	4 h
Amyloglucosidase	340 U/mL	5 mL	60 °C	6.0	1 h
Proteinase	340 U/mL	5 mL	60 °C	4.3	30 min

* The solution was 50 mmol/L maleic acid buffer.

**Table 2:** Mobile phase used for the detection of Avns by HPLC.

Time	Mobile Phase A: 0.1% Acetic Acid Water	Mobile Phase B: Acetonitrile
0.01 min	80%	20%
4 min	80%	20%
14 min	40%	60%
15 min	40%	60%
21 min	80%	20%
25 min	80%	20%
25.01 min	80%	20%

**Table 3:** Hydrolysis rates of oats at different time points.

Time (min)	0 Day	3 Day	7 Day	14 Day	21 Day	Control (White Bread)
0	24.060 ± 0.263 ^e^	23.422 ± 0.152 ^d^	18.513 ± 0.263 ^c^	13.692 ± 0.152 ^b^	9.738 ± 0.401 ^a^	26.684 ± 0.550
5	25.664 ± 0.264 ^e^	24.371 ± 1.150 ^d^	21.405 ± 0.455 ^c^	13.955 ± 0.402 ^b^	9.397 ± 0.304 ^a^	28.275 ± 0.401
10	25.225 ± 2.113 ^d^	24.195 + 0.152 ^d^	21.231 ± 0.152 ^c^	15.182 ± 0.152 ^b^	11.051 ± 0.152 ^a^	31.053 ± 0.664
30	28.591 ± 0.264 ^e^	26.867 ± 0.152 ^d^	21.536 ± 0.152 ^c^	16.881 ± 0.548 ^b^	13.692 ± 0.402 ^a^	35.185 ± 0.264
60	30.930 ± 0.152 ^d^	30.702 ± 0.264 ^d^	24.084 ± 0.456 ^c^	17.320 ± 0.152 ^b^	14.173 ± 0.403 ^a^	36.101 ± 0.403
120	30.526 ± 0403 ^d^	30.495 ± 0.152 ^d^	24.474 ± 0.304 ^c^	18.425 ± 0.152 ^b^	15.139 ± 0.264 ^a^	36.411 ± 0.401
180	35.186 ± 0.546 ^e^	31.084 ± 0.152 ^d^	28.068 ± 0.152 ^c^	19.828 ± 0.263 ^b^	15.796 ± 0.547 ^a^	38.099 ± 0.402

Different lowercase letters in each line indicate significant differences among groups (*p* ≤ 0.05).

**Table 4:** HI and eGI of SSF oats.

Day	HI	eGI
0	0.855 ± 0.009 ^e^	40.220 ± 0.005 ^e^
3	0.832 ± 0.015 ^d^	40.207 ± 0.008 ^d^
7	0.677 ± 0.002 ^c^	40.122 ± 0.001 ^c^
14	0.497 ± 0.004 ^b^	40.023 ± 0.002 ^b^
21	0.400 ± 0.002 ^a^	39.970 ± 0.001 ^a^

Different lowercase letters in each column indicate significant differences among groups (*p* ≤ 0.05).

## Data Availability

The original contributions presented in this study are included in the article. Further inquiries can be directed to the corresponding author.

## Abbreviations

The following abbreviations are used in this manuscript:

SSF	Solid-state fermentation
eGI	Estimated glycemic index
HI	Hydrolysis index
Avns	Avenanthramides
Avn A	Avenanthramide A
Avn B	Avenanthramide B
Avn C	Avenanthramide C
Avn D	Avenanthramide D
DF	Dietary fiber
TDF	Total dietary fiber
IDF	Insoluble dietary fiber
SDF	Soluble dietary fiber
GABA	γ-Aminobutyric acid
TPC	Total phenolic content
M1	M. purpureus wild strain
PDA	Potato dextrose agar medium
DNS	3,5-Dinitrosalicylic acid
PAL	Phenylalanine ammonia-lyase
TAL	Tyrosine ammonia-lyase
4CL	4-Coumarate: coenzyme A ligase
HHT	Hydroxyanthranilate N-hydroxycinnamoyltransferase
GAD	Glutamic acid decarboxylase
LAB	Lactic acid bacteria
RS	Resistant starch
RDS	Rapidly digestible starch
SDS	Slowly digestible starch
TFC	Total flavonoid content
XN	Xylaria nigripes
